# Round the Hospitals

**Published:** 1918-09-14

**Authors:** 


### ROUND THE HOSPITALS.

The date of the meeting of the College of Nursing to consider the formation of a London Centre has been fixed for Wednesday, September 25, at 7 P.M., at the College of Ambulance, Vere Street, W. The meeting promises to be of considerable interest, and amongst the questions to be discussed is that of a suitable clubroom for College members. Members should therefore attend on September 25 in full force to hear the report of the progress made, and to nominate officers and committees for the coming year. The Hon. Sir Arthur Stanley, G.B.E., will be amongst the speakers. It is hoped that advantage may be taken of the occasion to contradict officially the rumour which is being circulated that the College is registering members of Voluntary Aid Detachments who are not trained nurses. The rumour is without foundation, and all members of the College and those who have its interests and those of the nursing profession at heart should strenuously deny it.

ROUND THE HOSPITALS—(*continued*).

The non-competitive examination to qualify for election to the three sister-tutor studentships of 100 guineas each to enable trained nurses, who are members of the College of Nursing, to take a course of instruction for one year at King's College for Women, was held on September 3 at King's College and at the offices of the Scottish Board of the College of Nursing at Edinburgh. Of the twelve candidates, eleven represented England and one Scotland. Each candidate had to take two papers, one professional and the other on general knowledge, and these papers, together with the candidates' hospital records and references, are now being considered by the Council of the College. The announcement of the results will be awaited with interest.

---

The latest Local Centre of the College of Nursing is that for Newcastle-on-Tyne, which was formally constituted at a meeting held in the Royal Victoria Infirmary on August 30. The counties of Northumberland and Durham are to be included in the Centre, and the subscription was fixed at 2s. 6d. for this year and 5s. for succeeding years. Miss E. C. Brown, R.R.C., matron of the Royal Victoria Infirmary, Newcastle-on-Tyne, was appointed chairman and local representative; Miss Amour, matron of the Royal Infirmary, Sunderland, Hon. Treasurer; and Miss Mary Herbert, 3 St. Helen's Terrace, Low Fell, Gateshead, with Miss Flora Jones, Royal Victoria Infirmary, Newcastle-on-Tyne, joint honorary secretaries. General and executive committees were also elected. All members of the College in both counties are requested to send in their names and addresses to one of the honorary secretaries and to state which branch of nursing they are engaged in.

---

A syllabus of the post-graduate course of lectures offered to nurses in connection with the Liverpool Centre of the College of Nursing is now being prepared. The course, which is exceptionally varied and full of interest, will commence early in October, and continue till the end of April. It will offer opportunities of a unique kind to members of the nursing profession desirous of keeping abreast of the times in a period when nursing is undergoing many fruitful developments.

---

We published a short time back a letter from a correspondent expressing regret that so far the heroic services of the nursing sisters on hospital ships had received no recognition, and we then intimated that these brave deeds had not been overlooked, although formal recognition had been delayed. We have now the pleasure to furnish a list of matrons, sisters, and nurses whose names have been brought to the notice of the Secretary of State for War for valuable services rendered on the occasion of the sinking or damage by enemy action of hospital ships and transports. Miss A. E. Cashin, R.R.C., has been awarded a Bar to the Royal Red Cross for devotion to duty on the occasion of damage by enemy action to the hospital ship on which she was serving. The list of "mentions" comprises:—*Q.A.I.M.N.S.*—Matrons E. A. Dowse, R.R.C.; Mrs. Mitchell, R.R.C. *Q.A.I.M.N.S.R.*—Matrons A. E. Cashin, R.R.C.; Miss Corder; Sisters W. G. Coombs, B. M. E. Hesketh, A. Meldrum, A.R.R.C., A. Oakley; Staff-nurses H. M. G. Brooks, J. Macdonald, M. Marr, E. A. Walton, A.R.R.C. *T.F.N.S.*—Matron E. Robertson, R.R.C.; Assistant Matron M. Cowie, A.R.R.C.; Sisters L. M. Fieldsend, A. Taylor, E. Williams; Staff-nurses A. Campbell, E. Jennings, M. Lyall, H. O. Spencer. *B.R.C.S.*—Nurses W. M. Brazil, C. G. Brown, K. Coleman, E. Halford, D. A. Hannam, G. M. Johnson, Mrs. Lee, Mrs. Lewis, Nurse Lovibond, M. Mountain, M. D. Northern. Special Military Probationer, B. G. Mattison. *V.A.D.*—M. Prichard.

---

In the report of the Nightingale Fund for 1917 we note that the number of probationers in the school has shrunk from eighty to fifty-nine. In other respects the year was full of promise. The inauguration of a weekly gymnastic class (non-compulsory) for probationers is a move forward of vital importance. It is under the direction of Miss Needham, teacher in the physico-therapeutic department, and the nurses have shown enthusiasm. In the preliminary school a full-sized teaching model, as used in the United States training-schools, has been installed; the first of the kind to be available for instructing English nurses and of real value. Miss Montgomery, matron of the Middlesex Hospital, with assistance undertook the final nursing examination in theory and practice. Five nurses attained the standard required for the gold medal—viz., Edith Mary Heale, E. Katerine Servetopulos, Theodora West-Watson, Muriel Balmain, Gladys Hillyers. The gold, silver, and bronze medals were awarded to the first three in order of merit. The work in the wards on the civil side of the hospital continues to be most strenuous, and the management express thanks to the sisters "who have so ably carried on this work and the teaching of the nurses without the help of senior staff nurses."

---

This is the first year in which it has been possible to judge the full effect of the new scheme of instruction inaugurated by Miss Lloyd Still in 1914, under a sister-tutor, and the result, as shown by the keen competition for the gold medal, is very satisfactory indeed. Lest any may believe that the head is over-cultivated at St. Thomas's to the neglect of other forces in the individual, Miss Lloyd Still emphasises in her report the danger of laying too great a stress on lectures and examinations whereby practical nursing in the wards might suffer —a danger she is careful to try to avoid. We are

**ROUND THE HOSPITALS**—*(continued)*.

inclined to believe that her system, because it leads to real mastery of the subjects taught, must relieve probationers of that pursuing worry about lectures and examinations which is likely to vitiate their work. By evenly distributing the book work over a term of years, the nightmare rush preceding the final examination is abolished and that ordeal loses half its terrors.

A NEW light has been thrown on the subject of nurses' voting privileges by the announcement made at the last Quarterly Court of the London Hospital that the nurses in this establishment who occupy separate rooms are entitled to have their names on the register. As there appears to be some difference of opinion on this matter, some Registration officers holding, as we mentioned a short time ago, that separate rooms do not constitute a claim to a vote unless a week's notice has to be given before a change of room takes place, it is highly important that some general agreement should be reached on this legal point. It is manifestly unfair that nurses in some parts of London should be disqualified, while others, housed under similar conditions, are admitted to the poll.

THE rapid progress of aviation has not failed to make a concomitant psychological impression on the organisers of its Services. The R.A.F. is a new branch of the forces, untrammelled by traditions, and is therefore in a favourable position for the introduction of novelties, and it is not surprising, therefore, to learn that among the attractions offered to candidates for the R.A.F. Nursing Service is the granting of commissioned honorary rank. Matrons-in-Chief will have major's rank, matrons and superintending sisters that of captain; sisters will be lieutenants, and staff nurses second-lieutenants. Their uniforms will be of R.A.F. blue. The Matron-in-Chief will wear a frock faced and braided, a cape, and a bonnet of the same material. Matrons and sisters will wear similar uniform unbraided, a cape, and a bonnet of the same material. Superintending sisters and sisters will wear becoming three-cornered hats of blue straw in summer and of blue felt in winter. Light-blue trench coats may be worn in suitable weather.

THE effect of this innovation will be watched with interest by an interested and sympathetic public. In principle it should raise the status of the nursing staff, enhance their self-respect, and facilitate administration; but in practice much will depend on the selection of suitable material for the experiment—it is conceivable that such conditions and surroundings may attract those more interested in the social advantages than in the care of sick and wounded. The selection of candidates, therefore, will require circumspection. A proportion of the nursing staff has already been transferred to the Force from the Army and from the Navy, and others have been lent from these Services. For the remainder of the Service the Matron-in-Chief can no doubt rely on the pick of the newly trained women out of the major training-schools. These are the women who are most acceptable in the positions analogous to that of Second Lieutenant in the older sister Services. The attitude of the officers of the Force is expectant and somewhat sceptical. They are strong and healthy, and do not anticipate sickness or injury, and are quite satisfied with the matron and the sisters. They hope that military rank will not result in any change of type. A great responsibility thus devolves upon the new nurses of the R.A.F. Upon their response to the higher mission with which they have been entrusted will to a great extent depend their own future development and progress, as well as that of their sisters in other branches of the Army.

THE report of Miss Cummins, lady superintendent and matron of the Royal Infirmary, Liverpool, on the training-school, though brief, forms a very interesting feature in the annual report of that hospital for 1917. Of the thirty-five probationers who entered, eight proved unsuitable—a proportion of 22.8 per cent. This is much the same as at the Nightingale School, where nineteen out of eighty proved unsuitable—a percentage of 23.7. During the year twenty-five completed three years' training, and twenty-one completed their fourth year. Of the latter seven were promoted to sisters' posts, ten left to do military nursing, two for district nursing, and two to become sisters in other hospitals. Miss Crichton, matron's assistant, who had been ten years in the service of the infirmary, was appointed matron of the Dumfries and Galloway Royal Infirmary, and one of the sisters was appointed matron of a military hospital. There are at present sixty-one members of the nursing staff doing naval or military work at home and abroad. The standard of training appears, however, to be well maintained, and lectures have been given by eight members of the medical staff "under difficult circumstances." The school of massage has had a very successful year.

IT has been brought to our notice that the North Staffordshire Infirmary, in which the recent handsome rise of salaries has taken place, commented on in our issue of August 24, has no nursing institute attached to it. The Staffordshire Nursing Institute is an entirely separate organisation under a very able matron, Miss Wolseley-Lewis. It has always lent cordial assistance to the infirmary whenever possible in any times of shortage of nursing staff, and bears a high reputation. The sisters of the Infirmary, after five or more years' completed service, are now in receipt of £62 a year, probably a maximum salary so far as provincial institutions are concerned.

GREAT efforts are being made by the authorities of the Metropolitan Asylums Board to meet the grave shortage of nurses in the institutions under

**ROUND THE HOSPITALS—(*continued*).**

their control. The number of probationers now required has risen to 600, and some hundreds of ward orderlies for the domestic staff. We understand that the new bonus of the Metropolitan Asylums Board will bring their minimum pay for untrained women up to £38 a year, with a yearly rise; this in addition to board, lodging, uniform, and laundry. The rate of the bonus will be announced shortly, and will no doubt stimulate recruiting for the Service, which is going on through the local Labour Exchanges at the M.A.B. Headquarters on the Embankment. Matrons of voluntary hospitals and Poor-Law infirmaries usually have a large connection which brings them in candidates either through local or other sources owing to the standing and attractions of the training-school, and a good class of women is much more likely to be drawn into the Service in this way by desire to be trained under a particular matron than when they are directed to a central office. It would seem desirable that a great stimulation of effort should take place in the matron's department in every one of the institutions so sorely handicapped for want of staff.

The Canadian Association of Nursing Education, which met in June, had before it matters of great importance to the future of nursing in the Dominions, principally that of a standard curriculum for nurses. The main difficulty in regard to a fixed standard was clearly stated by Miss E. I. Johns, of Winnipeg: "The small country hospitals are going to maintain training-schools for some years to come, whether we like it or not. It is our duty, as nurse educators, to see that the teaching done in these schools is as good as we can make it." It is believed that the way to success lies in securing trained nurse instructors in these hospitals under the matron, in regular and sympathetic inspection, and in affiliation. To clear the ground of preconceived theories and see facts as they are is to have gone a long way already towards rectifying the defects of the present system.

To supply the deficiencies in the American Army Nursing Service, Dr. Goldwater advocates, in the August number of the *American Journal of Nursing*, the formation of a corps of "non-professional, voluntary war nursing aides," composed of women belonging to the leisured class, "the only labour reserves the country possesses." He thinks the United States could easily obtain and give short training in civil hospitals to 25,000 nurses' assistants before the end of this year, and gives reasons for adopting this plan in opposition to the proposal to train nurses in war hospitals. More than 10,000 women have already taken a standard course of short training under the American Red Cross. The system is one which, with some admitted drawbacks, has answered uncommonly well in this country, but it is not so easy as Dr. Goldwater thinks to ensure that nursing aides will not hereafter take up private nursing on the strength of their war experiences.

The Household Orderly Corps which Mrs. C. S. Peel, C.B.E., under the auspices of the Women's Industrial Council, proposes to form is intended to organise for domestic help a band of women trained in various branches of housework. They are to live off the premises and receive no meals, and their services are to be available by the hour. It is estimated that to attract a good class of worker the rate of pay should be 30s. a week, a rate which works out at 6d. an hour, reckoning an average of sixty hours' work a week. If the cost of housing accommodation, board, laundry, uniform, and wages for the domestic staff be worked out it will be found in many private houses to amount to considerably more than 30s. a head. In institutions the average is a good deal lower, and the hours of work expected from the domestic staff often amount to about seventy in the week. Nevertheless, if Mrs. Peel succeeds in her enterprise certain hospitals which are now in great straits for domestic help would probably be very glad to make use of her Household Orderly Corps. It is a well-thought-out attempt to deal with a pressing problem, and we hope to hear more about it. We advise all interested in this project to write to Mrs. Boyd Dawson, Women's Industrial Council, 6 York Buildings, Adelphi, W.C.

We have pleasure in recommending some timely recipes for using carrots, so rich in food value and at present so plentiful. The recipes are sold, price 6d., for the benefit of the Hampstead War Hospital Supply Depôt. Carrot and lemon marmalade is a useful preparation, the proportion being 5 lb. of carrots, 3 lb. of sugar, 5 lemons. This makes nearly 10 lb. of good firm marmalade. The carrots are scrubbed and peeled, boiled till tender in just sufficient water to cover them, and mashed through a wire sieve. The lemons are cut into thin slices and soaked for twelve hours in a soup-plate just covered with water, while the pips are soaked separately in half a cup of water. Boil carrot purée, lemon slices, and sugar for half an hour. Add jelly from pips and tie down in jars. If not firm too much water has been used, and a pint packet of Chivers' lemon jelly may be added.

**☛ It is our invariable practice to pay for all items of news which we may publish. The minimum payment is 5s. Paragraphs of about twenty lines in length—that is to say, of 150 words or less—are preferred. Contributions should be addressed to The Editor, The Hospital, 28 and 29 Southampton Street, Strand, London, W.C. 2, and, to be in time for the current week's issue, should reach him by the first post on Mondays.**

**For the Nurse's Voice: The Power of the College of Nursing, by "Ierne," see p. 520; Matrons, and Nurse Agitators, see p. 525 Matrons' and Sisters' Appointments, p. 526.**

